# Trends and Contextual Factors Related to Obesity-Related Legislation in the Republic of Korea (2006–2024)

**DOI:** 10.3390/nu18172848

**Published:** 2026-08-31

**Authors:** Yuri Lee, Hyun-Young Shin

**Affiliations:** 1 Department of Health and Medical Information, Myongji College, Seoul 03656, Republic of Korea; wittyyurilee@gmail.com; 2 Department of Public Health, Environments and Society, London School of Hygiene & Tropical Medicine, London WC1H 9SH, UK; 3 Department of Family Medicine, Seoul St. Mary’s Hospital, College of Medicine, The Catholic University of Korea, Seoul 06591, Republic of Korea

**Keywords:** obesity legislation, public health policy, Republic of Korea, multiple streams framework, child nutrition

## Abstract

Objectives: The objective of this paper is to analyse trends in obesity-related legislation in the Republic of Korea (2006–2024) and examine political, institutional, and ideational factors observed in relation to legislative outcomes. Methods: A retrospective policy analysis of 53 obesity-related bills identified through systematic searches of the National Assembly Legislative Information System and independently coded by two researchers. Quantitative trends were examined by policy domain and instrument type, and qualitative analysis applied Kingdon’s Multiple Streams Framework, the Ideas–Interests–Institutions (3I) model, and Policy Instrument Theory. Results: Legislative attention increased unevenly across the study period. Sixteen bills (30.2%) achieved a favorable legislative outcome, comprising direct passage or incorporation into an enacted committee alternative. Regulatory instruments dominated, followed by educational and institutional tools; fiscal measures were rare. Legislative activity clustered in 2013 and 2017–2019, while later proposals increasingly addressed school, digital, and post-pandemic concerns. Conclusions: Korea’s experience shows both progress and fragmentation in obesity governance. Child-focused framing and institutional feasibility were recurrent features among enacted measures, whereas fiscal and structural proposals were less frequently enacted. The findings highlight the importance of considering regulatory, educational, and fiscal approaches within a comprehensive intersectoral policy framework.

## 1. Introduction

Obesity has become a major public health concern worldwide, contributing to the growing burden of non-communicable diseases and healthcare costs [1]. In the Republic of Korea, where obesity prevalence was historically relatively low, the burden has increased substantially in recent years. National data show that adult obesity (body mass index ≥ 25 kg/m^2^) increased from 30.6% in 2013 to 38.4% in 2023, with particularly high prevalence among men (49.8% compared with 27.5% among women) [2]. Childhood and adolescent excess weight has also become an increasing concern; in a 2022 national adolescent survey, 29.1% were categorized as overweight based on BMI [3]. These trends are concerning given the well-established association of obesity with diabetes, cardiovascular disease, and certain cancers [4], and have contributed to the growing policy attention to obesity prevention and management in Korea [5].

Globally, there is increasing recognition that policy and environmental changes—not just individual behavior change—are essential to tackle obesity [6]. The World Health Organization (WHO) and other international bodies have called for comprehensive approaches including legislation, regulation, and fiscal measures to promote healthy diets and physical activity [7]. For example, the WHO Commission on Ending Childhood Obesity recommends implementing taxes on sugar-sweetened beverages and restricting the marketing of unhealthy foods to children as part of a “whole-of-government” response [8]. Such interventions, often termed structural or upstream policies, can help reshape obesogenic environments (e.g., food systems, schools, urban design) that encourage excessive calorie intake and sedentary lifestyles [6]. There is also a strong economic rationale: the OECD estimates that every dollar spent on preventing obesity can yield up to six dollars in economic returns by reducing future healthcare costs and productivity losses [9]. Consequently, many countries have begun to adopt legal and regulatory measures—from junk food advertising bans to front-of-pack nutrition labels and healthy school meal standards—in efforts to curb obesity [10].

Republic of Korea’s policy response to obesity has evolved in this global context. Until the mid-2000s, obesity was not explicitly targeted by national legislation, though related issues were addressed piecemeal through various laws on health promotion, food safety, school health, and sports [11]. The National Health Promotion Act (1995) provided a broad foundation for lifestyle disease prevention (including provisions on nutrition and physical activity), and other statutes such as the School Meals Act and School Health Act set nutritional standards and health services in schools. However, as obesity rates climbed—especially among children—the Republic of Korea saw a surge of legislative interest in more focused obesity prevention policies starting around 2007 [12]. Notably, advocacy by experts and civil society, including the Korean Society for the Study of Obesity, elevated obesity as an issue of national importance rather than just personal responsibility [13]. This shift in ideas and problem framing coincided with increased legislative attention to obesity prevention and management.

This study provides a comprehensive analysis of obesity-related legislation in the Republic of Korea from 2006 to 2024, focusing on two main questions: (1) What have been the patterns and outcomes of legislative efforts to address obesity (e.g., how many bills were introduced, in which areas, and with what success rate)? (2) What contextual factors were observed in relation to the adoption or non-adoption of these policy proposals? To answer these, we adopt a multidisciplinary theoretical lens that combines the Policy Instrument Theory with Kingdon’s Multiple Streams Framework (MSF) and the 3I framework (Ideas, Interests, Institutions). This integrated approach enables both a descriptive mapping of policy instruments and domains, and an interpretive analysis of political and ideational factors related to legislative decisions. By examining the Republic of Korea’s experience in depth, we aim to contribute to the global understanding of how evidence and politics converge in obesity policy-making. The findings carry implications for future public health law interventions—highlighting the importance of timing, problem framing, stakeholder alignment, and institutional context in translating public health needs into effective legislation.

## 2. Methods

### 2.1. Study Design

We conducted a retrospective policy analysis of obesity-related legislation in the Republic of Korea from 2006 through May 2024, covering the 17th to the 21st National Assembly. The full dataset comprised 53 eligible bills, all of which were included in the annual trend analyses. The study included both quantitative and qualitative components. On the quantitative side, we tracked all relevant bills introduced during these five legislative terms and compiled data on their frequency, policy domains, instrument types, and legislative outcomes such as passage or failure. On the qualitative side, we examined the content of each bill along with supporting documents, including bill preambles, committee reports, parliamentary debates, and news articles, in order to understand the context and rationale behind each proposal. We then applied the selected theoretical frameworks to interpret emerging patterns in the data. This mixed-methods approach combined descriptive statistical analysis with process tracing to capture both the measurable trends and the underlying political dynamics of policy development.

### 2.2. Data Collection

Relevant bills were identified through the National Assembly Legislative Information System [14]. The search covered the 17th to the 21st National Assemblies (May 2004–May 2024) and was conducted between January and February 2026, with the final search completed on 7 February 2026. Separate keyword searches were performed in the “Reason for Proposal and Major Contents” field using the exact Korean terms “비만” (obesity) and “영양” (nutrition). Because the two keyword searches produced overlapping records, the search results were combined, and duplicates were removed before eligibility assessment. No eligible bill was identified in 2004 or 2005; therefore, the analytic period began in 2006.

Two researchers independently reviewed the candidate bills and screened nutrition-related bills for substantive relevance to obesity policy by examining each bill’s title, reason for proposal, major contents, and substantive provisions. Records were included when they explicitly addressed obesity prevention or management or had a substantive, non-incidental connection to obesity-related nutrition policy, food environments, physical activity, or relevant health-system measures. Accordingly, the dataset included both obesity-specific bills and broader legislation when the latter had a substantive, non-incidental connection to nutrition, the food environment, or other obesity-related policy areas. Such broader laws were treated as indirectly relevant obesity-related legislation rather than as obesity-specific laws. For example, the Food Industry Promotion Act was retained as broader food-system legislation relevant to nutrition and the food environment, while the Special Act on Imported Food Safety Management was retained as broader food-safety legislation relevant to the safety and quality of foods and health functional foods; neither was classified as an obesity-specific law. Records were excluded when the keywords appeared only incidentally without substantively relevant policy provisions, or when they were resolutions, temporary budget riders, or other items that did not propose substantive policy changes. Disagreements were resolved through discussion until consensus was reached. Following de-duplication and expert screening, 53 unique bills were included in the final analysis.

For each included bill, we recorded essential information such as its title, date of introduction, legislative session, lead sponsor and party affiliation, committee referral, main provisions, and final legislative outcome. A complete bill-level list, including key metadata and legislative outcomes, is provided in Supplementary Table S1.

### 2.3. Variables and Categorization

Each bill was coded by legislative session, year of introduction, policy domain, policy instrument, and legislative outcome. Legislative sessions from the 17th to the 21st National Assembly were used to examine changes across legislative terms. Annual trend analyses included all 53 eligible bills introduced from 2006 through the May 2024 study cutoff. Each bill was assigned to a single primary domain based on its main objective.

During data extraction, bills were initially coded into five substantive subdomains: child and school nutrition, general nutrition and food environment, physical activity and exercise promotion, healthcare and treatment, and multi-sectoral strategy. For consistency in analysis and reporting, these subdomains were subsequently consolidated into three broad policy domains used throughout Section 3 and Supplementary Table S1: (1) Health (Public Health and Nutrition), (2) Education (School-Based), and (3) Sports/Physical Activity.

Following the Policy Instrument Theory, the dominant instrument was classified as regulatory, economic or fiscal, educational or informational, or institutional or framework-based. Regulatory instruments imposed mandates or restrictions such as advertising limits or BMI checks. Economic or fiscal tools included taxes, subsidies, and insurance incentives. Educational or informational measures involved public campaigns and school-based programs, while institutional or framework instruments established new organizations or coordination mechanisms. When a bill contained multiple policy domains or instruments, the primary category was selected according to its stated main objective, principal policy mechanism, and predominant operative provisions; secondary elements were not double-counted in the main analyses. This classification enabled analysis of the frequency and legislative outcomes of each policy type.

Legislative outcomes were coded as directly enacted/passed, incorporated into an enacted committee alternative, not enacted, or pending at the study cutoff. For the main analysis, directly enacted bills and bills incorporated into enacted committee alternatives were combined as favorable legislative outcomes. Enacted bills passed the National Assembly and became law, whereas non-enacted bills were either rejected or expired at the end of the term without a vote. In calculating favorable legislative outcome rates, rejected and lapsed bills were treated as not enacted.

### 2.4. Data Analysis

We analyzed the number of bills by year and legislative session to identify temporal trends. Enactment rates were calculated to assess whether legislative success improved or declined over time. Policy domains were cross-tabulated with instrument types to explore associations, such as whether child-focused bills were more likely to employ regulatory measures.

For each enacted law and selected major failed bills, we summarized their key provisions and assessed their political and social context. Qualitative materials were selected when they directly addressed the rationale, deliberation, stakeholder positions, or implementation context of an included bill. Official legislative materials—including bill proposals, committee review reports, and parliamentary deliberations—were prioritized, while government documents, news articles, and stakeholder materials were used as supplementary contextual sources. The materials were reviewed thematically for evidence of problem recognition, policy framing, stakeholder interests, political support or opposition, and institutional constraints, and these findings were subsequently interpreted using the MSF and 3I frameworks.

Major failed bills were selected for in-depth qualitative analysis when they represented substantively important policy approaches, including comprehensive obesity governance, fiscal intervention, or structural regulation, and when sufficient legislative documentation was available to reconstruct the policy process. To reduce subjectivity, case selection was based on these predefined criteria rather than on the perceived importance of individual bills.

To interpret the findings, we applied the MSF and the 3I model. Each major legislative episode was mapped against the MSF streams—problem, policy, and politics—while identifying relevant ideas, stakeholder interests, and institutional dynamics.

This study did not require ethical approval because it relied exclusively on publicly available legislative materials and did not involve human participants. By integrating systematic data collection with theory-informed interpretation, the analysis provides a structured foundation for examining contextual factors related to obesity-related legislation.

### 2.5. Analytical Framework and Approach

This study employed an integrated analytical framework combining Policy Instrument Theory, Kingdon’s Multiple Streams Framework (MSF), and the Ideas–Interests–Institutions (3I) model to interpret the political and institutional dynamics of obesity-related legislation in Korea.

First, according to Policy Instrument Theory, public policies are implemented through different tools or mechanisms that governments can use to influence behaviour and achieve policy goals. These instruments are often categorised as regulatory (“sticks”), economic or fiscal (“carrots”), and informational or educational (“sermons”) tools [15]. In the context of obesity prevention, these include: (i) regulatory measures restricting unhealthy food marketing or mandating nutrition labelling; (ii) fiscal instruments such as taxes on sugar-sweetened beverages or subsidies for healthy school meals; and (iii) educational campaigns promoting healthy diets [16,17].

Second, to understand when and why certain obesity policies progressed through the legislative process, we applied Kingdon’s Multiple Streams Framework (MSF) [18,19]. MSF conceptualises policy change in terms of the convergence of three independent “streams”—Problem, Policy, and Politics—which may coincide with opening of a “policy window” for reform. The framework allows identification of key focusing events (e.g., rising childhood obesity, the COVID-19 pandemic) and the timing of legislative momentum.

Third, to explore why some policy ideas gained traction while others failed, we integrated the Ideas–Interests–Institutions (3I) framework [10,20]. This model examines how ideas (beliefs and problem framing), interests (stakeholder motivations and power), and institutions (rules, administrative structures, and legacies) relate to policy processes and outcomes.

**Figure 1:**
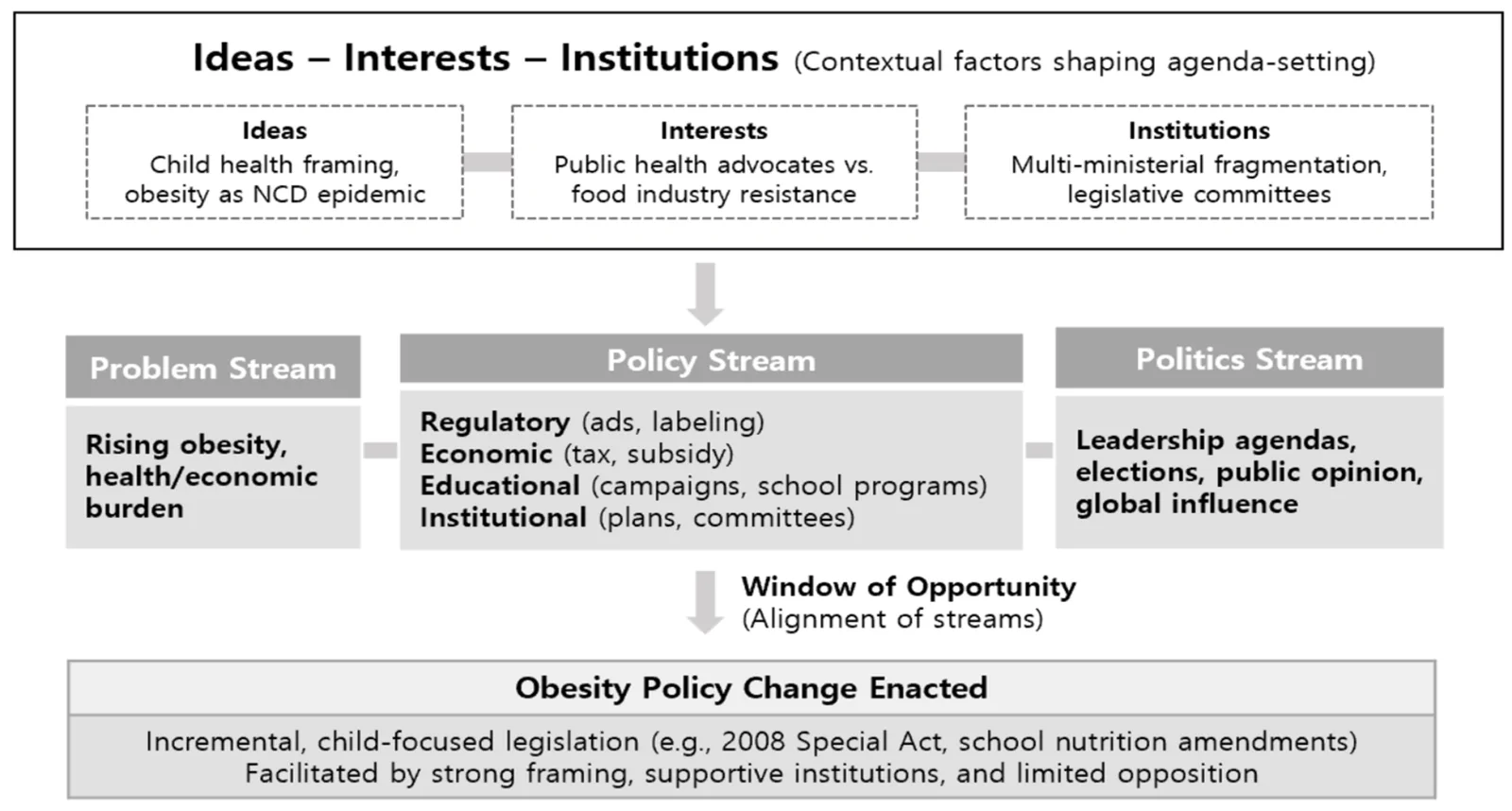
Integrated conceptual framework combining Policy Instrument Theory, Multiple Streams Framework (MSF), and 3I Framework.

Finally, drawing on recent scholarship emphasising the complementarity of these models [21], we developed a combined analytical lens. Policy Instrument Theory was used to categorise legislative strategies, MSF to map temporal agenda-setting dynamics, and the 3I framework to interpret the political economy underlying each major legislative episode. Figure 1 (conceptual model) illustrates how these contextual dimensions were examined in relation to major legislative episodes.

## 3. Results

### 3.1. Legislative Trends and Passage Rates (2006–2024)

Legislative attention to obesity in the Republic of Korea increased unevenly over the study period. The exact annual proposal counts in the final dataset were: 2006, 2; 2007, 4; 2008, 2; 2009, 4; 2010, 1; 2011, 3; 2012, 1; 2013, 7; 2014, 1; 2015, 2; 2016, 1; 2017, 5; 2018, 3; 2019, 6; 2020, 2; 2021, 5; 2022, 2; and 2023, 2; no new eligible bill was identified by the May 2024 cutoff. The highest annual count was seven in 2013, followed by six in 2019. Cumulatively, 53 bills were included, of which 16 achieved a favorable legislative outcome—10 were directly passed and six were incorporated into enacted committee alternatives—yielding an aggregate favorable-outcome rate of 30.2% (Table 1).

Table 1 summarizes bill introduction and outcomes by legislative session. The 17th Assembly cohort contained six eligible bills, including two introduced in 2006 and still active in 2007; two achieved favorable outcomes. The 18th and 19th Assemblies included ten and eleven bills, respectively, with two and one favorable outcomes. The 20th Assembly included fifteen bills, four of which achieved favorable outcomes. In the 21st Assembly, eleven bills were introduced and seven achieved favorable outcomes, representing the highest rate among the five assemblies.

The laws enacted illustrate incremental but significant policy change. These included the Special Act on Children’s Dietary Life Safety Management (2008), which established “Green Food Zones” and restricted child-targeted junk food advertising; amendments to the National Health Promotion Act (2010, 2019) that introduced calorie labeling in chain restaurants and created the Health-Friendly Workplace Certification scheme; the School Health Act amendments (2011, 2021, 2023) that mandated BMI monitoring and later required digital health programs for students; the School Meals Act amendment (2021) introducing fruit and vegetable snack programs; and the 2023 expansion of advertising restrictions to digital platforms. Other enacted measures, such as provisions in the School Physical Education Promotion Act (2020), strengthened oversight of school measures to promote students’ physical fitness and physical activity.

It is noteworthy that while many of the successful laws focused on specific environments such as schools or workplaces, several attempts at comprehensive “Obesity Prevention and Management Acts” were also made in the 18th, 19th, and 20th Assemblies. These proposals sought to establish an overarching legal framework mandating national master plans, inter-ministerial committees, and long-term strategies. However, none of these bills passed, and broader structural proposals were less frequently enacted than narrower, incremental measures. Thus, enacted laws were predominantly targeted or incremental in scope, whereas broader framework legislation was not enacted during the study period. This pattern is examined further in the contextual analysis below. Periods of intensified legislative activity and their associated contextual drivers are summarized in Table 2.

### 3.2. Policy Domains and Instrument Types

Table 3 categorizes all 53 bills by their primary policy domain and dominant policy instrument. Child and school nutrition was the largest clearly identifiable themed category after broader public-health/nutrition bills, accounting for about one in five proposals in our dataset. This domain encompassed the 2008 Special Act on Children’s Dietary Life Safety Management and its subsequent amendments, as well as initiatives to regulate snack foods in school zones, improve school meal nutrition, and restrict the sale of soft drinks in educational settings. South Korea’s school meal system was established through the School Meals Act in 1981 and gradually expanded to achieve universal coverage by 2003. Elementary schools were the first to be included in 1993, followed by middle schools in 1994 and high schools in 1997. Since the 2010s, the introduction of free meal programs has integrated nutritional welfare into the broader education and social policy framework. The dominance of child-focused legislation reflects both a strategic and ethical priority, as children are recognized as a vulnerable population and a politically sympathetic focus for public health intervention. This pattern mirrors global trends in which governments have prioritized child health through marketing restrictions, healthy school meal programs, and environmental changes that foster healthy behaviors [22].

Bills addressing the general nutrition and food environment targeted population-level determinants such as dietary quality, labelling, and economic incentives. Proposals included front-of-pack nutrition labelling, taxation of sugar-sweetened beverages, and strengthening national food standards. For example, a 2018 proposal to tax sugary drinks resembled international models such as those implemented in Mexico and the United Kingdom, which demonstrated measurable reductions in consumption and obesity risk [23,24]. However, these proposals were not enacted. Available legislative and contextual materials raised concerns regarding fiscal burden, implementation feasibility, and potential consumer impact, but did not permit attribution of non-enactment to any single stakeholder group. During the same period, Korea continued to rely primarily on non-fiscal approaches, including nutrition-labeling and educational measures. Korea did not rely solely on voluntary nutrition labeling: mandatory labeling applied to designated processed-food categories, including multiple beverage categories [25,26]. Nationally representative estimates of retail coverage or the independent effects of voluntary labeling were not identified.

Approximately one in ten bills focused on promoting physical activity, including community sports facilities, enhanced school physical education, and public campaigns. The 2020 School Physical Education Promotion Act amendment strengthened institutional oversight of measures to promote students’ physical fitness and physical activity; the original bill also identified rising obesity-related concerns among younger students as part of its policy rationale.

Another set of proposals treated obesity as a medical issue, seeking National Health Insurance coverage for counselling and treatment and the establishment of public obesity clinics. These faced technical and fiscal scrutiny regarding clinical consensus and budget impact and were not enacted. Internationally, however, recognition of obesity as a chronic, relapsing disease has strengthened [27].

Finally, comprehensive multi-sectoral bills sought to institutionalize national coordination through inter-ministerial committees, periodic master plans, and population monitoring. Three such “Basic Act on Obesity Prevention and Management” bills were introduced in 2015, 2019, and 2021; earlier versions expired at term’s end and the most recent remained pending. Repeated failure underscores the political and institutional difficulty of sustaining cross-sectoral reforms that demand long-term coordination and financial commitment.

Table 4 summarizes the frequency of policy instruments employed across all 53 obesity-related bills included in the analysis. Regulatory measures such as restrictions, mandates, and labelling requirements were the most common. Six of the 19 regulatory proposals achieved favorable legislative outcomes. Information and education-based instruments, which included awareness campaigns and curriculum revisions, accounted for roughly one quarter of all proposals but had similarly low enactment rates.

Economic and financial instruments—such as taxes, subsidies, and incentives—were the least common, representing fewer than one in five bills. Three bills classified primarily as economic/financial instruments achieved favorable legislative outcomes: the Food Industry Promotion Act Bill (2007), the Dietary Education Support Act Amendment Bill (2017), and the National Health Promotion Act Amendment Bill (Committee Alternative) (2019). These measures involved financial support, subsidies, or incentives within broader policy frameworks; none constituted a standalone obesity-related tax or other independent fiscal measure.

### 3.3. Summary of Enacted Legislation

Sixteen bills achieved favorable legislative outcomes during the study period, including direct enactment and incorporation into enacted committee alternatives (Table 5). Early acts such as the 2008 Special Act on Children’s Dietary Life Safety Management and the 2009 Dietary Education Support Act established foundational measures for healthy eating and child protection, while later amendments to the National Health Promotion Act and School Health Act expanded preventive efforts to workplaces and schools. From 2018 onward, new provisions expanded workplace wellness, dietary education, school health, and school-based physical activity initiatives. More recent reforms—such as the 2023 expansion of digital media advertising restrictions and the 2024 School Health Act amendment introducing digital obesity monitoring—reflect a shift toward technology-enabled, system-level prevention. Overall, these enactments illustrate Korea’s gradual transition from targeted nutrition policies to comprehensive, multi-sectoral approaches emphasizing structural and child-focused obesity prevention.

### 3.4. Contextual Factors Related to Legislative Outcomes: MSF and 3I Analysis

Table 6 maps the enacted obesity-related laws in Korea to the Ideas–Interests–Institutions (3I) analytical framework, illustrating contextual factors observed alongside different legislative outcomes. Across the enacted laws, recurring patterns included normative policy framing, identifiable stakeholder interests, and institutional pathways, although the strength of documentary evidence varied across cases. Early laws, such as the 2008 Children’s Dietary Life Safety Act and the 2009 Dietary Education Support Act, reflected prominent child-health, nutrition, and dietary-education framing. Subsequent acts—including the 2010 National Nutrition Management Act and the 2019 National Health Promotion Act amendment—illustrated evidence-oriented nutrition policy and the use of established administrative structures. More recent legislation, such as the 2023 Children’s Dietary Life Safety Act amendment and the 2024 School Health Act amendment, incorporated digital and institutional developments in obesity-related policy. Collectively, these cases suggest that policy framing, stakeholder context, and institutional feasibility were recurrent contextual features associated with legislative progression; however, the analysis does not establish causal relationships between these factors and legislative outcomes.

### 3.5. Population Obesity Trend, 2007–2024

**Figure 2:**
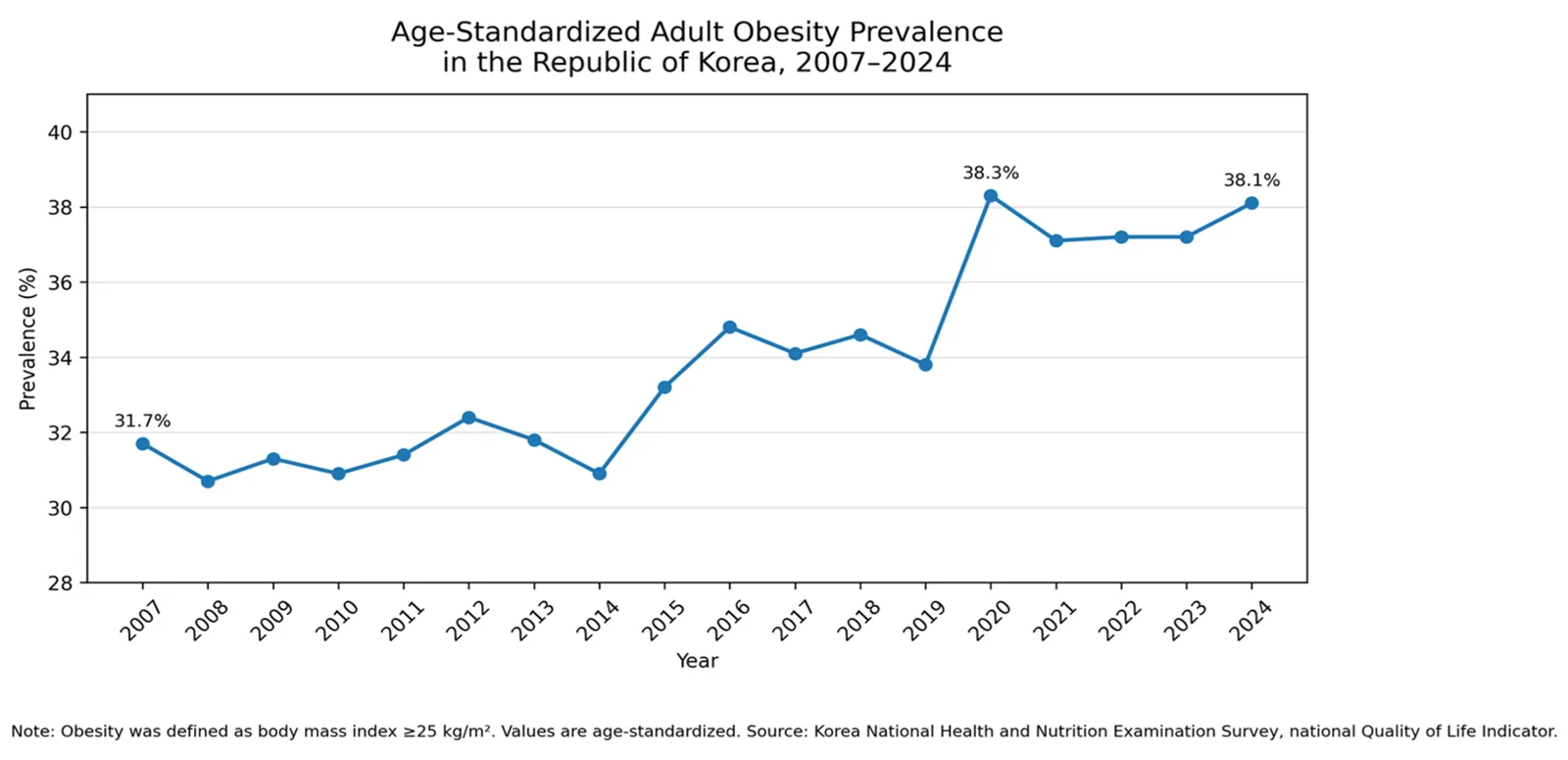
Age-standardized adult obesity prevalence in the Republic of Korea, 2007–2024. Source: Korea National Health and Nutrition Examination Survey (KNHANES).

Adult obesity prevalence increased from 31.7% in 2007 to 38.1% in 2024, with a marked increase to 38.3% in 2020 and persistently elevated levels thereafter (Figure 2) [28]. This population-level trend provides contextual evidence and should not be interpreted as a causal effect of individual legislative measures.

## 4. Discussion

Our analysis reveals a dynamic but complex trajectory for obesity-related legislation in the Republic of Korea. Between 2006 and 2024, obesity evolved from a peripheral concern into a mainstream policy issue, as reflected in the steady increase in legislative proposals and the broadening of policy scope. Despite this growing attention, the overall passage rate of about 30% underscores that translating concern into enforceable law remains challenging. Korea’s experience parallels the global pattern whereby obesity is widely acknowledged as a critical public health crisis, yet governments often struggle to implement comprehensive, enforceable measures. Comparative policy studies have identified political, economic, and institutional factors that may complicate comprehensive obesity policy [29]. The Korean findings show similar contextual challenges, although the present analysis does not establish their causal effects on legislative outcomes.

A key finding is the strong legislative emphasis on child-centered interventions. Most successful bills focused on protecting and promoting child health, from the 2008 Children’s Dietary Life Safety Act to recent school-based initiatives. This focus aligns with international recommendations, including those of the WHO Commission on Ending Childhood Obesity and the Lancet Commission, which advocate childhood obesity prevention as an entry point for broader NCD control [22,29]. Measures in children’s settings—such as school meals, media advertising restrictions, and nutrition education—may be comparatively politically feasible because child health provides a salient and broadly acceptable policy frame. Korea’s early adoption of junk food advertising restrictions and their subsequent expansion to digital platforms indicate continued legislative attention to children’s dietary environments. This mirrors trends in other high-performing policy contexts such as Chile, where comprehensive food marketing restrictions and front-of-pack warning labels were associated with measurable reductions in sugary drink consumption [30]. Although Korea has opted for voluntary labeling schemes rather than mandatory warning labels, both approaches illustrate the strategic viability of framing child obesity as a collective social responsibility rather than an issue of individual choice.

Conversely, legislative efforts targeting adults or employing fiscal instruments were less frequently enacted. Their non-enactment may have reflected multiple considerations, including concerns about consumer burden, administrative feasibility, political acceptability, and competing economic interests. However, the documentary materials examined in this study do not allow the relative influence of these factors, including food-industry interests, to be determined. Korea’s experience with tobacco taxation suggests that broader political and social context may be relevant to the feasibility of fiscal health measures, although the present analysis cannot establish the independent effect of public acceptance or political support.

The evolution of ideas about obesity in the Republic of Korea illustrates a broader ideological and institutional transformation. Until the early 2000s, obesity was largely viewed as a matter of personal willpower or aesthetics rather than a health issue, and government efforts focused on promoting general lifestyle improvement. Over time, international discourse—shaped by the WHO and OECD—and domestic advocacy reframed obesity as a medical and societal problem requiring policy intervention [31,32]. National insurance coverage for obesity treatment remained limited for many years, with metabolic/bariatric surgery first becoming reimbursable in 2019 for patients with severe obesity, marking a substantive policy shift toward recognizing obesity as a disease and a target for publicly funded treatment [33]. No legislation specifically establishing a framework for anti-obesity pharmacotherapy was identified during the study period. However, the safety of appetite suppressants regulated under the Narcotics Control Act has been addressed through broader pharmaceutical safety regulation, including strengthened reporting and monitoring of prescribing and dispensing to prevent misuse and overuse [34]. In addition, the Ministry of Food and Drug Safety introduced specific safe-use guidelines for medical narcotic appetite suppressants in 2020 [35]. Thus, Korea’s response in this area has relied primarily on pharmaceutical safety regulation and professional guidance rather than obesity-specific legislation. Given concerns regarding inappropriate use for cosmetic weight loss, continued public and professional awareness of the appropriate use of anti-obesity medications remains important. These developments illustrate the coexistence of individual-responsibility and collective public-health framings in obesity policy [36,37]. Framing obesity in economic terms—for example, by emphasizing productivity and healthcare costs—may have provided an additional policy rationale beyond health considerations [31,38]. The continued use of voluntary and educational approaches also illustrates the diversity of policy instruments used in Korea [39,40].

Implementation studies have reported modest changes in selected school food environments, including reduced availability of soft drinks, although unhealthy foods remained available in many school stores [41]. Digital food marketing has also remained an important challenge for child-focused obesity policies [42]. Similarly, studies of mandatory calorie labeling have reported increased information availability but only modest changes in consumer behavior [43]. These findings suggest that legislative measures should be considered alongside complementary interventions and systematic evaluation of implementation and outcomes. Consistent with the national trend shown in Figure 2, the persistently elevated obesity prevalence suggests that legislative progress has not yet coincided with a reversal of population-level trends, underscoring the need for a broader mix of regulatory, educational, healthcare, and potentially fiscal measures. The OECD emphasizes that “a wide range of policies are needed to see results” in obesity prevention, combining regulatory, fiscal, and educational measures [9]. In this context, renewed debate over a sugar-sweetened beverage tax in Korea reflects growing recognition that voluntary and informational approaches alone are insufficient, and that fiscal policies represent an additional policy option that warrants consideration and evaluation in the Korean context [23,24]. The eventual health outcomes will depend on how these regulatory, educational, and fiscal strategies are combined and evaluated over time.

This study provides a comprehensive review of obesity-related legislation in South Korea but has several limitations. First, it examines only primary legislation—bills and laws deliberated by the National Assembly—while excluding subordinate regulations such as enforcement decrees and ministerial ordinances, which play a critical role in policy implementation and compliance. Second, although the search and screening procedures were systematically applied, relevant bills may have been missed if they did not explicitly refer to obesity or nutrition, and the assignment of primary policy domains and instruments involved some interpretive judgment despite independent review by two researchers. Third, the analysis relies mainly on documentary data and does not include interviews or surveys with policymakers, bureaucrats, or advocacy groups that could illuminate the political trade-offs, institutional dynamics, and framing strategies underlying legislative outcomes. Media and stakeholder sources were used as supplementary contextual evidence and may not have comprehensively captured all relevant perspectives. Fourth, categorizing complex legislative trajectories into discrete outcome categories necessarily simplified some aspects of the legislative process. We also did not conduct a systematic analysis of legislative outcomes by political party, government/opposition status, committee, or number of sponsors. Finally, this study focused on legislative activity and outcomes and did not assess implementation intensity or the causal effects of legislation on obesity or other population health outcomes.

Future research should therefore integrate qualitative perspectives and policy practice analyses to assess how statutory provisions are enforced in real contexts—for example, whether school food zones are adequately monitored or workplace wellness programs are sustained after certification. Comparative cross-national research would also be valuable, contrasting South Korea’s child-centered legislative progress with adult obesity policies in countries such as Japan or the United Kingdom to explore how political structures, cultures, and governance arrangements shape policy adoption and enforcement. As obesity trends continue to evolve—with stabilization in some groups and increases in others amid post-pandemic lifestyle changes—evaluating emerging tools such as digital health interventions in schools, algorithm regulation in food delivery platforms, and fiscal measures such as sugar taxes should form the next frontier of obesity policy research.

## 5. Conclusions

South Korea’s legislative experience from 2006 to 2024 shows a gradual expansion from child-focused and nutrition-related measures to broader health-promotion and institutional approaches, while many comprehensive and fiscal proposals were not enacted. The MSF and 3I analyses identified recurring contextual patterns involving problem framing, stakeholder context, and institutional feasibility; however, these patterns should not be interpreted as causal determinants of legislative outcomes. Legislation can provide a statutory foundation for obesity policy, but implementation, compliance, and population-health effects require separate evaluation. These findings highlight the importance of continued assessment of how legislative measures are implemented and how different policy approaches operate within Korea’s evolving obesity-policy context.

## Figures and Tables

**Table 1:** Number of obesity-related bills proposed and passed by legislative session (17th–21st National Assembly).

Legislative Session (National Assembly)	Proposed Bills (*N*)	Passed Bills (*N*)	Passage Rate (%)
17th (2004–2008)	6	2	33.3
18th (2008–2012)	10	2	20.0
19th (2012–2016)	11	1	9.1
20th (2016–2020)	15	4	26.7
21st (2020–2024)	11	7	63.6
Total	53	16	30.2

Note. The years in parentheses indicate the full term of each National Assembly. The analytic period for this study began in 2006, when the earliest eligible bills were identified.

**Table 2:** Periods of legislative activity in obesity-related bills and contemporaneous policy contexts.

Period/Year	Legislative Activity	Contemporaneous Policy Contexts
2006–2014	Generally low but uneven activity, with a peak of 7 bills in 2013	Early stage of obesity policy; mostly individual initiatives; limited public salience.
2015–2019	Fluctuating activity, increasing to 5 bills in 2017 and 6 bills in 2019	Global discourse, including the UN Decade of Action on Nutrition (2016–2025) and the WHO Commission on Ending Childhood Obesity (2016); growing framing of obesity as a national public health issue.
Around 2018	Renewed legislative attention within the 2017–2019 period	Domestic developments, notably the launch of the Second National Obesity Management Comprehensive Plan (2018), coincided with increased governmental attention to obesity management.
2021–2022	Legislative cluster of 7 bills over two years (5 in 2021 and 2 in 2022)	The legislative cluster occurred during the COVID-19 period, when concerns about school health, dietary behavior, and physical activity were prominent in public-health discussions.
Overall pattern	Bursts of legislative proposals occurred sporadically when policy windows opened	International policy agendas, national health plans, and the COVID-19 pandemic formed part of the broader policy context in which periods of legislative activity occurred.

**Table 3:** Distribution of obesity-related bills by policy domain and policy instrument type (*n* = 53).

Policy Domain	Information/ Education (*N*)	Regulatory (*N*)	Economic/ Financial (*N*)	Organizational/ Planning (*N*)	Total
Health (Public Health and Nutrition)	10	12	9	7	38
Education (School-Based)	3	4	0	3	10
Sports/Physical Activity	0	3	0	2	5
Total	13	19	9	12	53

**Table 4:** Frequency of policy instrument types in obesity-related bills (*n* = 53).

Policy Instrument Type	Proposed Bills (*N*)	Passed Bills (*N*)
Regulatory (e.g., restrictions, mandates)	19	6
Information/Education (campaigns, curricula)	13	3
Economic/Financial (taxes, subsidies, incentives)	9	3
Organizational/Planning (institutions, strategic plans)	12	4
Total	53	16

Note. Regulatory instruments were the most frequently used policy tools (19 of 53), followed by information/education, organizational/planning, and fiscal instruments. Despite their prevalence, only a small portion of regulatory proposals were enacted. Fiscal instruments remained least common, but several were incorporated into broader institutional or regulatory bills—for example, subsidies for healthy meals and funding allocations for local health promotion programs. The three successful economic/financial bills involved financial support, subsidies, or incentives within broader policy frameworks; no standalone obesity-related tax or other independent fiscal measure was enacted.

**Table 5:** Summary of passed obesity-related legislation in the Republic of Korea (2006–2024).

Year	Legislation	Primary Content Summary
2007	Food Industry Promotion Act	Promotes development of the domestic food industry and traditional food culture through government support (e.g., R&D, training, quality standards).
2008	Special Act on Children’s Dietary Life Safety Management	Establishes “Green Food Zones” around schools and restricts the sale and advertising of high-fat, high-sugar, low-nutrient foods to protect children’s health.
2009	Dietary Education Support Act	Institutionalizes national dietary education programs to improve nutritional awareness and traditional food culture, under Ministry of Agriculture’s auspices.
2010	National Nutrition Management Act	Requires a national basic plan for nutrition management every 5 years and annual local nutrition plans; establishes a Nutrition Policy Council and mandates various government-run nutrition programs and periodic nutrition surveys.
2015	Special Act on Imported Food Safety Management	Strengthens safety controls on imported foods (e.g., requires overseas facility registration, permits on-site inspections abroad, and implements import traceability and risk-based import inspections).
2018	Regional Public Health Act (Amendment)	Requires local public health centers to hire certified health educators, enhancing capacity for community health promotion and chronic disease (including obesity) prevention programs.
2018	Dietary Education Support Act (Amendment)	Launches a program for regular provision of fruits and vegetables as snacks to children and adolescents, to foster healthy eating habits and support domestic farmers (new Article 26-2).
2019	National Health Promotion Act (Amendment)	Introduces “Health-Friendly Company” certification and adds physical activity promotion as a funded national health project, to encourage workplace wellness and reduce obesity and related health burdens.
2020	School Physical Education Promotion Act (Amendment)	Strengthened oversight of school measures to promote students’ physical fitness and physical activity and introduced additional safeguards concerning school sports facilities and student-athlete protection.
2021	School Health Act (Amendment)	Establishes a systematic student health promotion framework: mandates a 5-year Basic Plan for Student Health by the Education Minister and annual implementation plans by provincial education offices; creates school health promotion centers to monitor student physical development and mental health.
2021	Dietary Education Support Act (Amendment)	Encourages use of local agricultural products in school meal programs by explicitly allowing schools to provide regionally produced fruits, vegetables, and related products, thereby promoting healthy diets and local food systems (added Article 26-2).
2021	Dietary Education Support Act (Amendment)	Expands the content of national dietary education to include information on the origin of agricultural and food products, to improve consumer awareness and healthy eating practices (Article 7 revised).
2023	Special Act on Children’s Dietary Life Safety Management (Amendment)	Expanded the role of the National Children’s Food Management Center (renamed as the Dietary Safety Management Institute) and extended advertising restrictions to online and streaming platforms.
2024	School Health Act (Amendment)	Enhanced the digital component of student health programs, integrating obesity monitoring and prevention measures through school-based digital platforms and remote health services.

Note. The table includes both obesity-specific legislation and broader laws with substantive indirect relevance to nutrition, food environments, food safety, physical activity, or other obesity-related policy areas; inclusion does not imply that all listed laws were enacted specifically for obesity control.

**Table 6:** Mapping of passed obesity-related bills to 3I framework dimensions.

Legislation (Year Passed)	Ideas (Framing and Evidence)	Interests (Stakeholder Alignment)	Institutions (Policy Process and Rules)
Food Industry Promotion Act (2007)	Economic development idea	Food-industry relevance	Executive-legislative cooperation
Children’s Dietary Life Safety Act (2008)	Protect child health norm	Child-health stakeholder concerns and industry considerations	Health Committee leadership
Dietary Education Support Act (2009)	Cultural/traditional diet values	Agricultural-sector relevance	Cross-ministry coordination
National Nutrition Management Act (2010)	Evidence-based nutrition policy	Expert-policy context	Institutionalized planning requirement
Imported Food Safety Act (2015)	Food safety risk awareness	Consumer-protection context	Legislative deliberation
Regional Public Health Act Amend. (2018)	Community health equity	Public health advocates	Administrative mandate feasibility
Dietary Education Support Act Amend. (2018)	Healthy eating habits for youth	Agricultural-sector relevance	Government funding approval
National Health Promotion Act Amend. (2019)	Workplace wellness ideology	Workplace stakeholders implicated in implementation	Policy committee compromise
School Physical Education Promotion Act Amend. (2020)	Student physical fitness and school physical activity	Education authorities and school stakeholders	Oversight by the Ministry of Education and superintendents of education
School Health Act Amend. (2021)	Comprehensive student health view	Educational-authority role	Institutional capacity built
Dietary Education Support Act Amends. (2021—two bills)	Nutrition knowledge emphasis	Agriculture and health-sector involvement	Legislated program expansion
Children’s Dietary Act Amend. (2023)	Child protection priority	Child-protection rationale for advertising restrictions	Regulatory agency empowerment
School Health Act Amend. (2024)	Digital health innovation and obesity monitoring	Education Ministry and local governments collaboration	Integration of digital health infrastructure in school system

Note. Stakeholder-related entries reflect positions or institutional roles documented in legislative or official materials; where direct evidence of explicit support or opposition was unavailable, neutral contextual descriptions were used.

## Data Availability

The original contributions presented in this study are included in the article and Supplementary Materials. The legislative data were derived from publicly available records of the National Assembly Legislative Information System. Further inquiries can be directed to the corresponding author.

## Supplementary Materials

The following supporting information can be downloaded at https://www.mdpi.com/article/10.3390/nu18172848/s1, Table S1. Bills Included in the Analysis of Obesity-Related Legislation in the Republic of Korea (*n* = 53); Table S2. Annual Distribution of Bills by Legislative Outcome, 2006–2024 (*n* = 53).
