# Supplementary material for: Trends and Contextual Factors Related to Obesity-Related Legislation in the Republic of Korea (2006–2024)
Source: Nutrients. 2026 Aug 31;18(17):2848. doi: 10.3390/nu18172848 (PMC13567622; doi:10.3390/nu18172848)
Supplement: Supplementary file 1 [file nutrients-18-02848-s001.zip › nutrients-4467374-supplementary.pdf]

Supplementary Table S1. Bills Included in the Analysis of Obesity-Related Legislation in the Republic of Korea (n = 53)

No.	Bill title	Bill No.	Year	National Assembly	Sponsor	Political party	Policy domain	Policy instrument	Final legislative outcome
1	Child Welfare Act Amendment Bill	Not reported	2006	17th	Yoo Jeong-bok	Grand National Party	Health (Public Health & Nutrition)	Regulatory	Expired at end of term (not enacted)
2	National Health Promotion Act Amendment Bill	Not reported	2006	17th	Ahn Sang-soo	Grand National Party	Health (Public Health & Nutrition)	Information/Education	Expired at end of term (not enacted)
3	National Health Promotion Act Amendment Bill	176218	2007	17th	Gong Seong-jin	Grand National Party	Health (Public Health & Nutrition)	Information/Education	Expired at end of term (not enacted)
4	Special Act on Safety Management of Children's Dietary Life Bill	176278	2007	17th	Baek Won-woo	Uri Party	Education (School-Based)	Regulatory	Passed with amendment (enacted)
5	Framework Act on National Nutrition Bill	177660	2007	17th	Kim Young-sun	Grand National Party	Health (Public Health & Nutrition)	Organizational/Planning	Expired at end of term (not enacted)
6	Food Industry Promotion Act Bill	177700	2007	17th	Kwon Oh-eul	Grand National Party	Health (Public Health & Nutrition)	Economic/Financial	Passed with amendment (enacted)
7	Special Act on Safety Management of Children's Dietary Life Amendment Bill	1800650	2008	18th	Ahn Hong-jun	Grand National Party	Education (School-Based)	Regulatory	Passed with amendment (enacted)
8	National Nutrition Management Act Bill	1801790	2008	18th	Son Sook-mi	Grand National Party	Health (Public Health & Nutrition)	Organizational/Planning	Passed with amendment (enacted)
9	School Physical Education Act Bill	Not reported	2009	18th	Ahn Min-seok	Democratic Party	Sports/Physical Activity	Information/Education	Rejected (not enacted)
10	Special Act on Safety Management of Children's Dietary Life Amendment Bill	Not reported	2009	18th	Kim Hyo-jae	Grand National Party	Education (School-Based)	Regulatory	Expired at end of term (not enacted)
11	Special Act on Safety Management of Children's Dietary Life Amendment Bill	Not reported	2009	18th	Ahn Hong-jun	Grand National Party	Education (School-Based)	Regulatory	Expired at end of term (not enacted)
12	National Health Promotion Act Amendment Bill	Not reported	2009	18th	Kim Jae-yoon	Democratic Party	Health (Public Health & Nutrition)	Organizational/Planning	Expired at end of term (not enacted)
13	National Health Insurance Act Amendment Bill	Not reported	2010	18th	Jeong Yang-seok	Grand National Party	Health (Public Health & Nutrition)	Economic/Financial	Expired at end of term (not enacted)
14	Special Act on Safety Management of Children's Dietary Life Amendment Bill	Not reported	2011	18th	Kim Geum-rae	Grand National Party	Education (School-Based)	Regulatory	Expired at end of term (not enacted)
15	Food Sanitation Act Amendment Bill	Not reported	2011	18th	Jeon Hyun-hee	Democratic Party	Health (Public Health & Nutrition)	Regulatory	Expired at end of term (not enacted)
16	Special Act on Safety Management of Children's Dietary Life Amendment Bill	Not reported	2011	18th	Lee Nak-yon	Democratic Party	Health (Public Health & Nutrition)	Regulatory	Expired at end of term (not enacted)
17	Special Act on Safety Management of Children's Dietary Life Amendment Bill	Not reported	2012	19th	Lee Chae-ik	Saenuri Party	Health (Public Health & Nutrition)	Economic/Financial	Expired at end of term (not enacted)
18	National Health Promotion Act Amendment Bill	1903851	2013	19th	Kim Jae-yoon	Democratic United Party	Health (Public Health & Nutrition)	Organizational/Planning	Expired at end of term (not enacted)
19	Higher Education Act Amendment Bill	Not reported	2013	19th	Moon Dae-sung	Independent	Health (Public Health & Nutrition)	Information/Education	Expired at end of term (not enacted)
20	National Health Promotion Act Amendment Bill	Not reported	2013	19th	Moon Dae-sung	Independent	Health (Public Health & Nutrition)	Economic/Financial	Expired at end of term (not enacted)
21	Framework Act on the Management of Charges Amendment Bill	Not reported	2013	19th	Moon Dae-sung	Independent	Health (Public Health & Nutrition)	Economic/Financial	Expired at end of term (not enacted)
22	Special Act on Imported Food Safety Management Bill	1905394	2013	19th	Kim Myung-yeon	Saenuri Party	Health (Public Health & Nutrition)	Regulatory	Passed with amendment (enacted)
23	National Nutrition Management Act Amendment Bill	Not reported	2013	19th	Min Byung-doo	Democratic Party	Health (Public Health & Nutrition)	Information/Education	Expired at end of term (not enacted)
24	Food Sanitation Act Amendment Bill	Not reported	2013	19th	Min Byung-doo	Democratic Party	Health (Public Health & Nutrition)	Regulatory	Expired at end of term (not enacted)
25	Maternal and Child Health Act Amendment Bill	Not reported	2014	19th	Oh Je-se	Democratic Party	Health (Public Health & Nutrition)	Information/Education	Expired at end of term (not enacted)

No.	Bill title	Bill No.	Year	National Assembly	Sponsor	Political party	Policy domain	Policy instrument	Final legislative outcome
26	Narcotics Control Act Amendment Bill	Not reported	2015	19th	Han Myeong-sook	New Politics Alliance for Democracy	Health (Public Health & Nutrition)	Regulatory	Expired at end of term (not enacted)
27	Food Sanitation Act Amendment Bill	Not reported	2015	19th	Kim Tae-won	Saenuri Party	Health (Public Health & Nutrition)	Regulatory	Expired at end of term (not enacted)
28	Special Act on Safety Management of Children's Dietary Life Amendment Bill	Not reported	2016	20th	Joo Seung-yong	People's Party	Health (Public Health & Nutrition)	Regulatory	Expired at end of term (not enacted)
29	Narcotics Control Act Amendment Bill	Not reported	2017	20th	Pyo Chang-won	Democratic Party of Korea	Health (Public Health & Nutrition)	Regulatory	Expired at end of term (not enacted)
30	Marathon Promotion Act Bill	Not reported	2017	20th	Ham Jin-gyu	Liberty Korea Party	Sports/Physical Activity	Information/Education	Expired at end of term (not enacted)
31	Dietary Education Support Act Amendment Bill	2007449	2017	20th	Kim Hyun-kwon	Democratic Party of Korea	Health (Public Health & Nutrition)	Economic/Financial	Passed with amendment (enacted)
32	Regional Public Health Act Amendment Bill	2008145	2017	20th	Lee Myung-soo	Liberty Korea Party	Health (Public Health & Nutrition)	Organizational/Planning	Incorporated into an enacted committee alternative
33	Act on the Installation and Use of Sports Facilities Amendment Bill	Not reported	2017	20th	Kim Seok-ki	Liberty Korea Party	Sports/Physical Activity	Organizational/Planning	Expired at end of term (not enacted)
34	Act on Guarantee of the Right to Health and Access to Medical Services for Persons with Disabilities Amendment Bill	Not reported	2018	20th	Park In-sook	Liberty Korea Party	Health (Public Health & Nutrition)	Information/Education	Expired at end of term (not enacted)
35	Dietary Education Support Act Amendment Bill	Not reported	2018	20th	Wi Seong-gon	Democratic Party of Korea	Health (Public Health & Nutrition)	Information/Education	Expired at end of term (not enacted)
36	National Health Promotion Act Amendment Bill	2017533	2018	20th	Yoon Il-gyu	Democratic Party of Korea	Health (Public Health & Nutrition)	Organizational/Planning	Incorporated into an enacted committee alternative
37	Food Publicness Support Act Bill	Not reported	2019	20th	Kim Jong-hoe	Party for Democracy and Peace	Health (Public Health & Nutrition)	Organizational/Planning	Expired at end of term (not enacted)
38	School Meals Act Amendment Bill	Not reported	2019	20th	Wi Seong-gon	Democratic Party of Korea	Education (School-Based)	Economic/Financial	Expired at end of term (not enacted)
39	Special Act on Safety Management of Children's Dietary Life Amendment Bill	Not reported	2019	20th	Lee Yang-soo	Liberty Korea Party	Health (Public Health & Nutrition)	Regulatory	Expired at end of term (not enacted)
40	National Health Promotion Act Amendment Bill (Committee Alternative)	2023155	2019	20th	Chair, Health and Welfare Committee	Committee-sponsored (N/A)	Health (Public Health & Nutrition)	Economic/Financial	Committee alternative passed (enacted)
41	National Health Promotion Act Amendment Bill	Not reported	2019	20th	Nam In-soon	Democratic Party of Korea	Health (Public Health & Nutrition)	Information/Education	Expired at end of term (not enacted)
42	Special Act on Safety Management of Children's Dietary Life Amendment Bill	Not reported	2019	20th	Won Yoo-chul	Liberty Korea Party	Health (Public Health & Nutrition)	Regulatory	Expired at end of term (not enacted)
43	School Physical Education Promotion Act Amendment Bill	2100180	2020	21st	Lim Oh-kyung	Democratic Party of Korea	Sports/Physical Activity	Organizational/Planning	Incorporated into an enacted committee alternative
44	Dietary Education Support Act Amendment Bill	2100656	2020	21st	Wi Seong-gon	Democratic Party of Korea	Health (Public Health & Nutrition)	Information/Education	Incorporated into an enacted committee alternative
45	Special Act on Safety Management of Children's Dietary Life Amendment Bill	Not reported	2021	21st	Cho Myung-hee	People Power Party	Health (Public Health & Nutrition)	Regulatory	Pending at study cutoff (May 2024)
46	School Health Act Amendment Bill	2107478	2021	21st	Kang Min-jung	Open Democratic Party	Education (School-Based)	Organizational/Planning	Incorporated into an enacted committee alternative
47	National Health Promotion Act Amendment Bill	2108406	2021	21st	Kang Byung-won	Democratic Party of Korea	Health (Public Health & Nutrition)	Economic/Financial	Pending at study cutoff (May 2024)
48	Dietary Education Support Act Amendment Bill (Committee Alternative)	2111686	2021	21st	Chair, Agriculture, Food, Rural Affairs, Oceans and Fisheries Committee	Committee-sponsored (N/A)	Health (Public Health & Nutrition)	Information/Education	Committee alternative passed (enacted)
49	School Health Act Amendment Bill (Committee Alternative)	2112208	2021	21st	Chair, Education Committee	Committee-sponsored (N/A)	Education (School-Based)	Organizational/Planning	Committee alternative passed (enacted)

No.	Bill title	Bill No.	Year	National Assembly	Sponsor	Political party	Policy domain	Policy instrument	Final legislative outcome
50	School Sports Promotion Act Amendment Bill	2117403	2022	21st	Jung Chan-min	People Power Party	Sports/Physical Activity	Organizational/Planning	Incorporated into an enacted committee alternative
51	Special Act on Safety Management of Children's Dietary Life Amendment Bill	2117893	2022	21st	Jung Choun-sook	Democratic Party of Korea	Education (School-Based)	Regulatory	Passed with amendment (enacted)
52	School Meals Act Amendment Bill	Not reported	2023	21st	Seo Dong-yong	Democratic Party of Korea	Education (School-Based)	Information/Education	Pending at study cutoff (May 2024)
53	Special Act on Safety Management of Children's Dietary Life Amendment Bill	Not reported	2023	21st	Lee Jae-jung	Democratic Party of Korea	Health (Public Health & Nutrition)	Regulatory	Pending at study cutoff (May 2024)

Notes. The year refers to the year of introduction. Policy domain and policy instrument indicate the primary category assigned according to each bill's principal legislative purpose and substantive provisions. Where a bill contained multiple components, the dominant policy objective was used. "Incorporated into an enacted committee alternative" indicates that a member-sponsored bill was not enacted independently, but its provisions were incorporated into a committee alternative that subsequently passed. "Pending at cutoff" indicates the status as of 29 May 2024. "Not reported" indicates that the bill number was unavailable in the source dataset. Source: National Assembly Legislative Information System.

Supplementary Table S2. Annual Distribution of Bills by Legislative Outcome, 2006–2024 (n = 53)

Year	Bills introduced	Directly enacted/passed	Incorporated into enacted committee alternative	Not enacted	Withdrawn	Pending at cutoff	Other/not reported	Favorable legislative outcome
2006	2	0	0	2	0	0	0	0
2007	4	2	0	2	0	0	0	2
2008	2	2	0	0	0	0	0	2
2009	4	0	0	4	0	0	0	0
2010	1	0	0	1	0	0	0	0
2011	3	0	0	3	0	0	0	0
2012	1	0	0	1	0	0	0	0
2013	7	1	0	6	0	0	0	1
2014	1	0	0	1	0	0	0	0
2015	2	0	0	2	0	0	0	0
2016	1	0	0	1	0	0	0	0
2017	5	1	1	3	0	0	0	2
2018	3	0	1	2	0	0	0	1
2019	6	1	0	5	0	0	0	1
2020	2	0	2	0	0	0	0	2
2021	5	2	1	0	0	2	0	3
2022	2	1	1	0	0	0	0	2
2023	2	0	0	0	0	2	0	0
2024	0	0	0	0	0	0	0	0
**Total**	**53**	**10**	**6**	**33**	**0**	**4**	**0**	**16**

Notes. Table S2 presents the annual distribution of all 53 eligible bills introduced from 2006 through the study cutoff of 29 May 2024. The outcome categories—directly enacted/passed, incorporated into an enacted committee alternative, not enacted, withdrawn, pending at cutoff, and other/not reported—are mutually exclusive. "Not enacted" includes bills rejected or expired at the end of a National Assembly term. "Favorable legislative outcome" is a derived indicator equal to directly enacted/passed plus incorporated into an enacted committee alternative; it should not be added to the mutually exclusive outcome columns.
